# Supplementary material for: Experiences of Testing Negative or Positive for HIV in Malawi, South Africa, and Zambia: A Cross-Sectional Study
Source: AIDS Behav. 2025 Oct 18;30(3):766–77. doi: 10.1007/s10461-025-04921-6 (PMC12988891; doi:10.1007/s10461-025-04921-6)
Supplement: Supplementary file 1 — Supplementary file1 (DOCX 16 kb) [file 10461_2025_4921_MOESM1_ESM.docx]

**Supplementary Table 1: Participants characteristics by gender, HIV test result and Country**

|  | **Malawi** | | | | **South Africa** | | | | **Zambia** | | | |
| --- | --- | --- | --- | --- | --- | --- | --- | --- | --- | --- | --- | --- |
|  | **Male** | | **Female** | | **Male** | | **Female** | | **Male** | | **Female** | |
|  | **Positive** | **Negative** | **Positive** | **Negative** | **Positive** | **Negative** | **Positive** | **Negative** | **Positive** | **Negative** | **Positive** | **Negative** |
|  | (N = 48) | (N = 93) | (N = 56) | (N = 127) | (N = 27) | (N = 58) | (N = 65) | (N = 239) | (N = 53) | (N = 124) | (N = 95) | (N = 157) |
| **Age,** median (IQR) | 31  (25, 43) | 28  (24, 33) | 30  (24, 40) | 27  (21, 35) | 39  (31, 47) | 32  (25, 40) | 30  (24, 37) | 25  (22, 32) | 36  (31, 42) | 25  (22, 30) | 29  (24, 40) | 26  (22, 33) |
| **Marital status,** n (%) |  |  |  |  |  |  |  |  |  |  |  |  |
| *Never married* | 13 (27) | 26 (28) | 6 (11) | 26 (20) | 15 (56) | 44 (76) | 57 (88) | 212 (89) | 9 (17) | 67 (54) | 27 (28) | 56 (36) |
| *Married* | 20 (42) | 60 (65) | 33 (59) | 85 (67) | 9 (33) | 13 (22) | 7 (11) | 23 (10) | 28 (53) | 56 (45) | 41 (43) | 87 (55) |
| *Divorced/separated/widowed* | 15 (31) | 7 (8) | 17 (30) | 16 (13) | 3 (11) | 1 (2) | 1 (2) | 4 (2) | 16 (30) | 1 (1) | 27 (28) | 14 (9) |
| **Highest level of education,** n (%) |  |  |  |  |  |  |  |  |  |  |  |  |
| *No schooling/primary* | 27 (56) | 39 (42) | 31 (55) | 58 (46) | 17 (63) | 28 (48) | 28 (43) | 50 (21) | 27 (51) | 59 (48) | 57 (60) | 52 (33) |
| *Secondary* | 20 (42) | 45 (48) | 20 (36) | 56 (44) | 8 (30) | 23 (40) | 30 (46) | 149 (62) | 25 (47) | 50 (40) | 32 (34) | 84 (54) |
| *Post-secondary* | 1 (2) | 9 (10) | 5 (9) | 13 (10) | 2 (7) | 7 (12) | 7 (11) | 40 (17) | 1 (2) | 15 (12) | 6 (6) | 21 (13) |
| **Employment status, n (%)** |  |  |  |  |  |  |  |  |  |  |  |  |
| *Formal employment* | 8 (17) | 7 (8) | 7 (12) | 4 (3) | 8 (30) | 21 (36) | 11 (17) | 38 (16) | 7 (13) | 11 (9) | 4 (4) | 13 (8) |
| *Informal employment* | 32 (67) | 62 (67) | 31 (55) | 77 (61) | 12 (44) | 18 (31) | 8 (12) | 33 (14) | 45 (85) | 75 (60) | 47 (49) | 72 (46) |
| *Unemployed* | 5 (10) | 12 (13) | 18 (32) | 36 (28) | 6 (22) | 16 (28) | 38 (58) | 124 (52) | 1 (2) | 30 (24) | 40 (42) | 53 (34) |
| *Student/Trainee* | 3 (6) | 12 (13) | 0 (0) | 10 (8) | 1 (4) | 3 (5) | 8 (12) | 44 (18) | 0 (0) | 8 (6) | 4 (4) | 19 (12) |
| **Food scarcity, n (%)** |  |  |  |  |  |  |  |  |  |  |  |  |
| *Never* | 18 (38) | 28 (30) | 19 (34) | 53 (42) | 14 (52) | 41 (71) | 38 (58) | 155 (65) | 12 (23) | 41 (33) | 41 (43) | 69 (44) |
| *Seldom* | 8 (17) | 26 (28) | 6 (11) | 25 (20) | 3 (11) | 6 (10) | 5 (8) | 22 (9) | 7 (13) | 23 (19) | 10 (11) | 11 (7) |
| *Sometimes* | 19 (40) | 33 (35) | 21 (38) | 35 (28) | 7 (26) | 10 (17) | 13 (20) | 57 (24) | 32 (60) | 56 (45) | 39 (41) | 66 (42) |
| *Often* | 3 (6) | 6 (6) | 10 (18) | 14 (11) | 3 (11) | 1 (2) | 9 (14) | 5 (2) | 2 (4) | 4 (3) | 5 (5) | 11 (7) |
| **Would have difficulty obtaining R 100/1000 Kwacha for medical treatment, n (%)** | 37 (77) | 63 (67) | 38 (68) | 88 (69) | 18 (67) | 28 (48) | 40 (62) | 118 (49) | 38 (72) | 86 (69) | 75 (79) | 117 (75) |
